# Real‐Life Comparison of Antivirals for SARS‐CoV‐2 Omicron Infection in Patients With Hematologic Malignancies

**DOI:** 10.1111/irv.13264

**Published:** 2024-03-11

**Authors:** Tommaso Francesco Aiello, Olivier Peyrony, Mariana Chumbita, Patricia Monzó, Carlos Lopera, Pedro Puerta‐Alcalde, Laura Magnano, Francesc Fernández‐Avilés, Genoveva Cuesta, Montse Tuset, Josep Mensa, Jordi Esteve, Maria Angeles Marcos, Alex Soriano, Carolina Garcia‐Vidal

**Affiliations:** ^1^ Infectious Diseases Department, Hospital Clinic of Barcelona‐IDIBAPS Universitat de Barcelona Barcelona Spain; ^2^ Emergency Department, Hôpital Saint Louis Assistance Publique‐Hôpitaux de Paris Paris France; ^3^ Department of Haematology, Hospital Clinic of Barcelona‐IDIBAPS University of Barcelona Barcelona Spain; ^4^ Microbiology Department, Hospital Clinic University of Barcelona, ISGLOBAL Barcelona Spain; ^5^ Department of Pharmacy, Hospital Clinic of Barcelona‐IDIBAPS University of Barcelona Barcelona Spain; ^6^ Facultat de Medicina i Ciències de la Salut Universitat de Barcelona Barcelona Spain

**Keywords:** COVID‐19, nirmatrelvir/ritonavir, Omicron, outcome, phenotypes

## Abstract

**Background:**

We aimed to describe a cohort of hematologic patients with COVID‐19 treated with antivirals early.

**Methods:**

Non‐interventional chart review study. Comparison of baseline characteristics and outcomes in high‐risk hematologic patients treated with remdesivir between December 2021 and April 2022 versus those treated with nirmatrelvir/ritonavir between May and August 2022.

**Results:**

Eighty‐three patients were analyzed. Forty‐two received remdesivir, and 41 nirmatrelvir/ritonavir. Patients with remdesivir were younger, vaccinated with lower number of doses, and received prior corticosteroids less frequently and sotrovimab, hyperimmune plasma and corticosteroids more often. Viral shedding median (IQR) duration was 18 (13–23) and 11 (8–21) days in the remdesivir and nirmatrelvir/ritonavir groups, respectively (*p* = 0.004). Median (IQR) Ct values before treatment were similar in both groups. Within 5 days of treatment, median (IQR) Ct values were 26 (23–29) and 33 (30–37) in the remdesivir and nirmatrelvir/ritonavir groups, respectively (*p* < 0.0001). All patients were hospitalized for remdesivir administration and only four (9.8%) in the nirmatrelvir/ritonavir group. The overall outcomes in this cohort of COVID‐19 patients with Omicron variant was good, as no patient needed oxygen or ICU admission. One patient in remdesivir group died from septic shock. No severe adverse event was recorded in both treatment groups.

**Conclusions:**

Patients with hematologic malignancies and non‐severe COVID‐19 who received nirmatrelvir/ritonavir experienced faster decrease in viral load and shorter viral shedding. Furthermore, besides the advantage of oral administration, nirmatrelvir/ritonavir administration reduced the need of hospital admission.

## 1 Introduction

**FIGURE 1:**
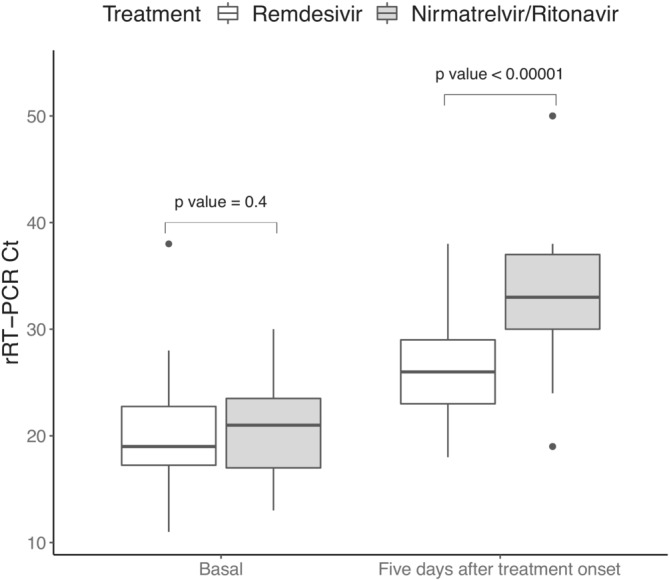
SARS‐CoV‐2 rRT‐PCR Ct shift after 5 days of treatment per antiviral regimen. White boxes refer to rRT‐PCR Ct of patients treated with remdesivir. Gray boxes refer to rRT‐PCR Ct of patients treated with nirmatrelvir/ritonavir. Box plots show the interquartile range (IQR). The black horizontal line inside de box indicates the median rRT‐PCR Ct. Whereas median (IQR) Ct values before antiviral treatment were similar (19 [17–23] in the remdesivir group and 21 [17–24] in the nirmatrelvir/ritonavir group), 5‐day median Ct values were 26 (23‐29) and 33 (30–37) in the in the remdesivir and nirmatrelvir/ritonavir groups, respectively (*p* < 0.0001). Black dots indicate low and high outliers.

**FIGURE 2:**
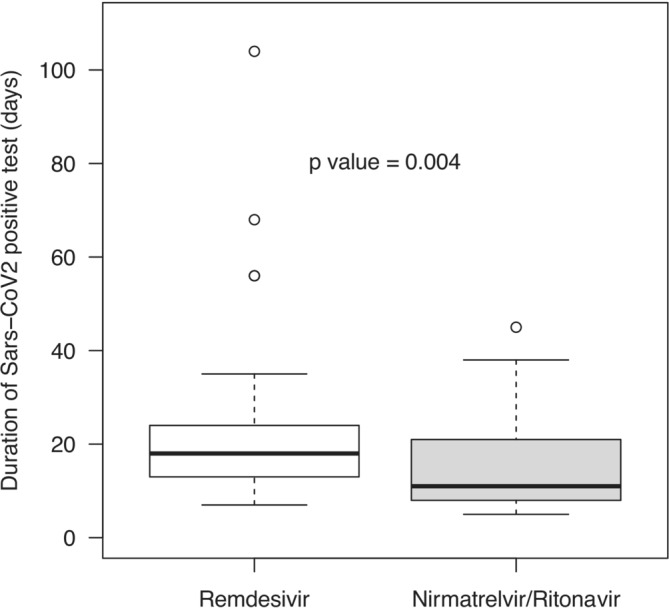
Median (IQ) duration in days of SARS‐CoV‐2 positive test per antiviral regimen. White box refers to duration in days of SARS‐CoV‐2 positive test of patients treated with remdesivir. Gray box refers to patients treated with nirmatrelvir/ritonavir. Box plots show the interquartile range (IQR). The black horizontal line inside de box indicates de median duration in days of SARS‐CoV‐2 positive test. White dots indicate low and high outliers.

Patients with hematologic malignancies and/or hematopoietic stem cell transplantation recipients have been significantly affected by COVID‐19: Morbidity and mortality rates have reached high figures [1, 2, 3, 4, 5]. Hematological patients undergoing anti‐CD38 and anti‐BCMA therapy experience impairment in immune cells such DCs, B cells, NK cells, and TFH cells, leading to an inability to generate adequate humoral and cellular responses to vaccination [6]. This suboptimal immune response to COVID‐19 vaccines results in both a more severe and prolonged course of SARS‐CoV‐2 infection and delays in management of severe underlying hematologic diseases [3, 7].

Therapeutic approaches for hematologic patients are especially challenging as these patients were either excluded from or poorly represented in major clinical trials, being optimal management strategies and treatments for COVID‐19 not well established in this population. Nevertheless, clinical experience suggests that antiviral therapies for COVID‐19, including remdesivir and sotrovimab, play a major role in improving outcomes [8, 9, 10]. Nirmatrelvir/ritonavir is an oral protease inhibitor authorized to treat mild‐to‐moderate COVID‐19 in patients at high risk for progression to severe COVID‐19. A randomized trial showed only 0.7% of patients who received nirmatrelvir/ritonavir—compared to 6.5% of patients with placebo—within 5 days of symptom onset were hospitalized until Day 28 post‐randomization, resulting in a relative risk reduction of 89% [11]. Yet, even then, patients with hematologic malignancies comprised less than 1% of this cohort. To our knowledge, data comparing the efficacy of different combined antiviral regimens in real‐life patients with hematologic malignancies and COVID‐19 are scarce [12].

In this study, we aimed to compare clinical characteristics, viral load, and outcomes in patients with hematologic malignancies and non‐severe Omicron variant COVID‐19 treated with remdesivir or nirmatrelvir/ritonavir.

## 2 Materials and Methods

### 2.1 Study Design and Patients

This observational cohort study included all consecutive patients with high‐risk hematologic malignancies early diagnosed with COVID‐19 (a cycle threshold [Ct] value lower than 28 in real‐time reverse transcriptase‐polymerase chain reaction [rRT‐PCR] or a Ct value ranging between 28 and 30, with positive sub‐genomic RNA) who received treatment with an antiviral regimen for SARS‐CoV‐2 Omicron variant infection at Hospital Clinic of Barcelona. The study period was between December 2021 and August 2022. We compared patients treated with remdesivir and those treated with nirmatrelvir/ritonavir. We included only COVID‐19 episodes of patients with no supplemental oxygen therapy at onset.

High‐risk hematologic malignancies were acute leukemia under intensive chemotherapy; lymphoma in either treatment or remission with rituximab therapy; multiple myeloma under active treatment; chronic lymphocytic leukemia with targeted therapies; high‐risk myelodysplastic syndrome in active treatment; and primary high‐risk myelofibrosis. Individuals receiving CAR T‐cell therapies and patients either within the first year of an allogenic hematopoietic stem cell transplantation (allo‐HSCT) or the initial 6 months of an autologous stem cell transplant (ASCT) were also deemed as high risk.

Using the electronic health records, we prospectively collected data for all patients included in the study. The outcomes of the study were need and length of hospital admission; need of ICU and/or mechanical ventilation; viral shedding duration; and mortality at the end of follow‐up. We also compared the evolution of patients' viral load after 5 days of antiviral treatment in both study groups.

The Institutional Ethics Committee of Hospital Clinic of Barcelona approved the study and, due to the nature of the observational data review, waived the need for informed consent from individual patients (HCB/2020/0273).

### 2.2 Microbiological Studies

All patients had a confirmed COVID‐19 diagnosis by either a rapid antigen test or rRT‐PCR performed on nasal and oropharyngeal throat swabs. An rRT‐PCR was performed 5 days after antivirals were administered to both treatment groups to assess the evolution of the Ct value and guide decision‐making processes regarding whether to continue supplemental therapies in patients with persistent viral shedding. For patients with a positive rRT‐PCR and Ct value > 26, viral sub‐genomic RNA (sgRNA) testing was performed. This was done as it better correlates with active viral replication [13]. To assess the persistence of viral shedding, a rapid antigen test was performed in patients receiving nirmatrelvir/ritonavir at the end of the 5‐day antiviral regimen if rRT‐PCR was not available. In addition, to determine the duration of viral shedding, all cohort patients with hematologic malignancies underwent further testing until negativization. Negativization was considered when rRT‐PCR showed either a Ct ≥ 40 or rRT‐PCR Ct > 26, with negative viral sub‐genomic RNA (sgRNA).

### 2.3 Antiviral Treatment Regimens

Beginning in December 2021, we administered antiviral regimens to all patients of our institution with high‐risk hematologic malignancies and a SARS‐CoV‐2 infection who presented either a Ct < 28 in rRT‐PCR or positive sgRNA at diagnosis and/or infection duration of ≤10 days of symptom onset. According to drug availability and drug interactions, those regimens were remdesivir‐based between December 2021 and April 2022 and nirmatrelvir/ritonavir‐based between May 2022 and August 2022. Seronegative patients with either a Ct < 26 or positive sgRNA also received convalescent plasma and/or sotrovimab. In patients who received nirmatrelvir/ritonavir and had a persistent Ct < 26 at the end of the 5‐day treatment, an additional course of remdesivir—with or without plasma and/or sotrovimab depending on serology—was administered. Figure S1a shows the flow chart of treatment algorithm. We assessed antiviral‐related adverse events and adverse events caused by a potential drug–drug interaction in both study groups.

### 2.4 Statistical Analysis

Categorical variables were described using the absolute number and percentage, while continuous variables were presented using the median and interquartile range (IQR). Categorical variables were compared using either a chi‐squared (χ^2^) test or Fisher's exact test when appropriate, and quantitative variables using the Mann–Whitney *U* test. Statistical significance was defined as a *p*‐value < 0.05. Analyses were performed using R v2.13.0.

## 3 Results

We included a total of 83 adults with hematologic malignancies. Of these, 63.9% were male, and 39.8% were older than 65 years. Most patients (94%) had received a prior COVID‐19 vaccine, and 15 (18.1%) have had a previous COVID‐19 episode. Only 44.6% had a positive serology at COVID‐19 diagnosis. During the study period, the Omicron BA.1 and BA.2 subvariants were predominant from December 2021 to Abril 2022, and the Omicron BA.4 and BA.5 subvariants were predominant from May 2022 to August 2022. Table 1 summarizes the main cohort characteristics. Most patients (90.4%) were symptomatic, presenting cough, pharyngitis, fever, and/or rhinorrhea. Median (IQR) duration of symptoms before positive test results was 0 (0–2) days, while median (IQR) time from diagnosis to treatment onset was 1 (0–4) days, 2 (1‐5) days in remdesivir‐based regimen, and 1 (0–3) days in nirmatrelvir/ritonavir‐based antiviral regimen.

**TABLE 1:** Main epidemiological and clinical characteristics of patients with hematologic malignancies and SARS‐CoV‐2 infection per treatment group.

	Total cohort (*N* = 83)	Remdesivir (*n* = 42)	Nirmatrelvir/ritonavir (*n* = 41)	Difference (95% CI)	*p*
Patient characteristics					
Age, in years, median (IQR)	62 (49–72)	58 (46–65)	67 (59–74)	−9.6 (−15.6 to −3.4)	0.005
Sex male, *N* (%)	53 (63.9)	27 (64.3)	26 (63.4)	0.9 (−22.2–24)	0.934
Baseline hematologic malignancy, *N* (%)					
Lymphoma	43 (51.8)	20 (47.6)	23 (56.1)	−8.5 (−32.3–15.4)	0.071
Acute leukemia	10 (12)	8 (19)	2 (4.9)	14.2 (−1.8–30.2)	0.030
Multiple myeloma	15 (18.1)	5 (11.9)	10 (24.4)	−12.5 (−31.3–6.3)	0.416
Chronic lymphocytic leukemia	5 (6)	3 (7.1)	2 (4.9)	2.3 (−10.4 to 14.9)	0.222
High‐risk myelodysplastic syndrome	6 (7.2)	3 (7.1)	3 (7.3)	−0.2 (−13.7–13.4)	0.837
Other^a^	4 (4.8)	3 (7.1)	1 (2.4)	4.7 (−6.8–16.2)	
Last/ongoing treatment before COVID‐19, *N* (%)					
Prior autologous hematopoietic stem cell transplantation	27 (32.5)	17 (40.5)	10 (24.4)	16.1 (−6.2–38.3)	0.120
Prior CAR T‐cell therapy	10 (12)	5 (11.9)	5 (12.2)	−0.3 (−16.7–16.1)	0.967
Prior corticosteroid use (3 months)	35 (42.2)	13 (31)	22 (53.7)	−22.7 (−45.8–0.4)	0.038
Prior chemotherapy (3 months)	63 (75.9)	30 (71.4)	33 (80.5)	−9.1 (−29.7–11.6)	0.336
Prior rituximab use (12 months)	29 (34.9)	15 (35.7)	14 (34.1)	1.6 (−21.4–24.5)	0.880
Other comorbidities, *N* (%)					
Arterial hypertension	28 (33.7)	13 (31)	15 (36.6)	−5.6 (−28.4–17.1)	0.587
Chronic heart disease	21 (25.3)	9 (21.4)	12 (29.3)	−7.8 (−28.9–13.2)	0.412
Chronic lung disease	7 (8.4)	4 (9.5)	3 (7.3)	2.2 (−12.1–16.5)	0.718
Diabetes mellitus	10 (12)	4 (9.5)	6 (14.6)	−5.1 (−21.5–11.3)	0.477
Chronic renal failure	10 (12)	5 (11.9)	5 (12.2)	−0.3 (−16.7–16.1)	0.967
Chronic liver disease	5 (6)	2 (4.8)	3 (7.3)	−2.6 (−15.2–10.1)	0.627
Solid neoplasm	5 (6)	1 (2.4)	4 (9.8)	−7.4 (−20–5.2)	0.191
Prior COVID‐19	15 (18.1)	0	15 (36.6)	−36.6 (−53.7 to −19.4)	0.990
Symptoms of SARS‐CoV‐2, *N* (%)					
Cough	47 (56.6)	22 (52.4)	25 (61.0)	−8.6 (−32.2–15.1)	0.430
Fever	32 (38.6)	17 (40.5)	15 (36.6)	3.9 (−19.4–27.2)	0.715
Pharyngitis	45 (54.2)	19 (45.2)	26 (63.4)	−18.2 (−41.7–5.3)	0.098
Rhinorrhea	29 (34.9)	9 (21.4)	20 (48.8)	−27.4 (−49.5 to −5.2)	0.010
Dyspnea	3 (3.6)	2 (4.8)	1 (2.4)	2.3 (−8.1–12.7)	0.577
Asymptomatic disease	8 (9.6)	7 (16.7)	1 (2.4)	14.2 (−0.4–28.9)	
Other clinical features, *N* (%)					
Prior COVID‐19 vaccination	78 (94)	39 (92.9)	39 (95.1)	−2.3 (−14.9–10.4)	0.666
Minimum of 3 doses of COVID‐19 vaccination	54 (65.1)	19 (45.2)	35 (85.4)	−36.6 (−58.1 to −15)	0.000
Positive SARS‐CoV‐2 serology at admission	37 (44.6)	19 (46.3)	18 (66.7)	−20.3 (−46.8–6.2)	0.102
Treatment, *N* (%)					
Remdesivir	45 (54.2)	42 (100)	3 (7.3)	92.7 (82.3–100)	0.993
Glucocorticoids	10 (12)	9 (21.4)	1 (2.4)	19 (3.3–34.7)	0.026
Hyperimmune plasma	27 (32.5)	23 (54.8)	4 (9.8)	45 (25–65)	0.000
Antibiotics	15 (18.1)	11 (26.2)	4 (9.8)	16.4 (−2.1–34.9)	0.060
Sotrovimab	10 (12)	9 (21.4)	1 (2.4)	19 (3.3–34.7)	0.027
Length of SARS‐CoV‐2 positive test (days), median (IQR)	15 (9–22)	18 (13–23)	11 (8–21)	7.7 (1.1–14)	0.004
SARS‐CoV‐2 positive test longer than 21 days since diagnosis, *N* (%)	21 (25.3)	13 (31)	8 (19.5)	11.4 (−9.5–32.4)	0.233
SARS‐CoV‐2 positive test longer than 28 days since diagnosis, *N* (%)	21 (25.3)	9 (21.4)	4 (9.8)	11.7 (−6.1–29.5)	0.152
Length of hospital stay (days), median (min–max)	5 (0–9)	9 (6–12)	6 (5–11)	3.5 (−2.9–10.1)	0.613

Abbreviations: Ct, cycle threshold; IQR, interquartile range; rRT‐PCR, real‐time reverse transcriptase polymerase chain reaction.

^a^ High‐risk myelofibrosis (one patient).

A total of 42 subjects received a remdesivir‐based antiviral regimen, while an additional 41 patients received a nirmatrelvir/ritonavir‐based antiviral regimen. Table 1 provides characteristic differences per treatment regimen. Patients in the remdesivir group were younger, more commonly had acute leukemia, had been less frequently vaccinated with three doses of the COVID‐19 vaccine, and had received prior corticosteroid treatments less often than those in the nirmatrelvir/ritonavir group. In terms of concomitant COVID‐19 treatments, patients in the remdesivir group more frequently received corticosteroids, hyperimmune plasma, and sotrovimab. A total of 29 (70.7%) patients in the nirmatrelvir/ritonavir group were diagnosed at onset by rapid antigen testing. The rRT‐PCR Ct at diagnosis was available for 61 patients (73.5%; 100% in the remdesivir group and 46.3% in nirmatrelvir/ritonavir group) and at Day 5, for 70 patients (84.3%, 88.1%, and 80.5%, respectively).

Figure 1 details the rRT‐PCR Ct before and after 5 days of antiviral treatment in both groups. Whereas median (IQR) Ct values before antiviral treatment were similar (19 [17–23] in the remdesivir group and 21 [17–24] in the nirmatrelvir/ritonavir group), 5‐day median Ct values were 26 (23–29) and 33 (30–37) in the remdesivir and nirmatrelvir/ritonavir groups, respectively (*p* < 0.0001). Figure 2 shows the median (IQR) duration of active SARS‐CoV‐2 infection as documented by rRT‐PCR, antigen testing, or viral sub‐genomic RNA (sgRNA). It was 18 (13–23) days in the remdesivir group and 11 (8–21) days in the nirmatrelvir/ritonavir group (*p* = 0.004). Positive test was documented after 45 days in a total of four (9.5%) patients from the remdesivir group and in none from the nirmatrelvir/ritonavir group.

In the remdesivir group, all 42 patients required hospitalization for antiviral parenteral administration; median (IQR) length of hospital stay was 9 (6–12) days. No patients received oxygen supplementation during hospital stay or required ICU admission. Among the 41 patients treated with nirmatrelvir/ritonavir, four (9.8%) required hospital admission. Two patients were admitted due to persistent rRT‐PCR, with a Ct < 26 at Days 14 and 20 since diagnosis. They received parenteral antiviral treatment (remdesivir for 5 days and hyperimmune plasma) and achieved negativization of SARS‐CoV‐2 rRT‐PCR at discharge. One patient required admission due to both cryptogenic organizing pneumonia and clinical suspicion of a bacterial superinfection and received corticosteroids and antibiotic treatment. One patient required admission due to persistent rRT‐PCR at Day 33 (Ct 18) since COVID‐19 diagnosis and received parenteral antiviral treatment (remdesivir for 5 days, hyperimmune plasma, and sotrovimab). This patient achieved negativization of SARS‐CoV‐2 rRT‐PCR at discharge. Median (IQR) length of hospital stay in these four patients were 6 (5–11) days. None of the four patients received oxygen supplementation during hospitalization or required ICU admission. Neither severe adverse events nor dangerous interactions were documented in patients on nirmatrelvir/ritonavir‐based nor remdesivir‐based regimens. Only one patient in the remdesivir group died at Day 36 since COVID‐19 diagnosis. This patient was in palliative care for end‐stage renal cell carcinoma and Hodgkin lymphoma. The cause of death was multiple organ failure due to septic shock, triggered by *Escherichia coli* infection.

## 4 Discussion

In this study involving high‐risk patients with hematologic malignancies (44.5% of whom received a hematopoietic stem cell transplantation or CAR T‐cell therapy) and COVID‐19, we found that subjects treated early with antiviral strategies had better outcomes, shorter viral shedding, and lower mortality than previous reported cohorts [2, 3, 8, 14]. There are some key factors to consider to understand our results. Firstly, all patients in our series were vaccinated, many of whom had already received three doses. Vaccines have been shown to improve the prognosis of patients with SARS‐CoV‐2 infection, even those who are immunocompromised [15, 16]. Secondly, the predominant variant during the study period was Omicron. This variant has been suggested to possibly have a not as severe clinical picture as other variants [17] and be associated less with pneumonia due to intrinsic characteristics [18]. Thirdly, early diagnosis—even with antigen self‐testing—allows for immediate antiviral treatment, which has been related with better outcomes [11, 19, 20, 21]. Finally, our protocol avoided the systematic use of dexamethasone—except in those patients with severe or critical COVID‐19—per current guidelines [22], and studies showed longer viral shedding and a higher mortality rate in patients with moderate and severe disease receiving dexamethasone alone or dexamethasone plus remdesivir [23, 24]. Only 22% of patients in our study received glucocorticoids during a COVID‐19 episode. This figure contrasts with data from other cohorts of patients with hematologic malignancies, in up to 42% of cases [25].

The use of remdesivir has been associated with both a decrease in mortality among non‐ventilated, immunocompetent adults with oxygen therapy and lower progression to mechanical ventilation and/or death [19, 26]. Of note, patients with hematologic malignancies were poorly represented in remdesivir clinical trial; thus, little information has been made available about the efficacy of this antiviral in such profiles. However, prescribing this antiviral requires hospitalization and/or complex organization to administer it intravenously. Our prior reported experience, consistent with other publications, documented that a 5‐day remdesivir regimen in high‐risk patients with hematologic malignancies was frequently associated with prolonged viral shedding and the risk of selecting SARS‐CoV‐2 variants containing mutations that escape from vaccines [27, 28, 29]. Therefore, some treatment recommendations for this type of patients warrant an extension of treatment to 10 days or a drug combination with monoclonal antibodies [30].

Nirmatrelvir/ritonavir is an oral antiviral with excellent results in treating immunocompetent patients with risk factors and mild‐to‐moderate COVID‐19 [11]. Some data suggest that this antiviral could be rather efficient in reducing SARS‐CoV‐2 viral load [31]. Our experience with a cohort of real‐life patients with hematologic malignancies receiving this antiviral is promising. The possibility of oral administration and ambulatory treatment have been important advantages when compared to other available antiviral strategies. In addition, the ability to suppress SARS‐CoV‐2 viral load in this population has been excellent, with a more rapid negative patient response than previously seen with other early antiviral strategies. Remarkably, whereas patients receiving nirmatrelvir/ritonavir in our cohort were high risk (56% had lymphoma, most receiving rituximab; 24%, multiple myeloma with several treatment lines; 24%, a stem cell transplantation; and 12%, CAR T‐cell therapy), the median length of SARS‐CoV‐2 positive testing after disease diagnosis was quite low (11 days). Furthermore, no patients had prolonged viral shedding for more than 45 days. This observation is of major importance, especially in terms of avoiding both the emergence of resistant variants and delays or interruptions of hematologic treatments. Moreover, no important side effects related to nirmatrelvir/ritonavir were documented in our cohort. These results support the use of this antiviral as first‐line treatment in high‐risk outpatients with hematologic malignancies. It is worth noting that nirmatrelvir/ritonavir has significant and complex drug–drug interactions, requiring an extensive review of concomitant medications prior to its prescription.

Our study has several limitations. First, our study is not a randomized, double‐blind study. This means that the populations that received one or the other antiviral strategy are somewhat different. However, our cohort did describe real‐life patients. Second, the Ct values were missing in some patients. Third, we are describing the experience of a single center. In contrast, all patients in the cohort were treated by the same physicians who had a uniform idea about ICU admission requirement, the moment for hospital discharge, and the use of other therapeutic strategies, such as corticosteroids. Finally, vaccination rates were similar in remdesivir‐based and nirmatrelvir/ritonavir‐based treatment. Nevertheless, only 46.3% and 66.7% of patients, respectively, presented positive SARS‐CoV‐2 serology at admission, probably due to the underlying hematological disease. This may have had an impact in viral clearance. The main strength of our study is useful results for the current management of patients with COVID‐19, especially given that it includes a cohort with mostly vaccinated patients with an Omicron variant infection. Our data support the benefit of oral early administration of antiviral treatment in high‐risk hematological patients in order to prevent prolonged viral shedding and need for hospital admission due to SARS‐CoV‐2 Omicron variant. This type of variant is the one circulating at the moment. Many studies to date discussing antiviral or anti‐inflammatory strategies are based on populations with variants that are no longer circulating or who are unvaccinated. This means that results from these studies are limited in utility [32].

As a conclusion, our study suggests that early administration of nirmatrelvir/ritonavir in patients with high‐risk hematological malignancies is an excellent option to promote a faster viral load decrease and improve COVID‐19 outcomes. In our patients, this oral antiviral helped prevent hospital admissions, without significant side effects, a need of mechanical ventilation or ICU admission, and/or death.

## Author Contributions

Conceptualization: Carolina Garcia Vidal and Tommaso Francesco Aiello. Data curation: Tommaso Francesco Aiello, Olivier Peyrony, Mariana Chumbita, Patricia Monzó, Carlos Lopera, and Pedro Puerta‐Alcalde. Formal analysis: Tommaso Francesco Aiello and Olivier Peyrony. Funding acquisition: Carolina Garcia Vidal and Alex Soriano. Investigation: Tommaso Francesco Aiello, Olivier Peyrony, Mariana Chumbita, Patricia Monzó, Carlos Lopera, Pedro Puerta‐Alcalde, Laura Magnano, Francesc Fernández‐Avilés, Genoveva Cuesta, Montse Tuset, and Carolina Garcia‐Vidal. Methodology: Carolina Garcia Vidal, Tommaso Francesco Aiello, and Olivier Peyrony. Project administration: Tommaso Francesco Aiello and Carolina Garcia Vidal. Software—programming: Tommaso Francesco Aiello and Olivier Peyrony. Supervision: Josep Mensa, Jordi Esteve, Maria Angeles Marcos, Alex Soriano, and Carolina Garcia‐Vidal. Writing—original draft: Tommaso Francesco Aiello and Carolina Garcia Vidal. Writing—review and editing: Josep Mensa, Jordi Esteve, Maria Angeles Marcos, Alex Soriano, and Carolina Garcia‐Vidal.

## Ethics Statement

The Institutional Ethics Committee of Hospital Clinic of Barcelona approved the study and due to the nature of the observational data review, waived the need for informed consent from individual patients (HCB/2020/0273).

## Conflicts of Interest

CG‐V has received honoraria for talks on behalf of Gilead Science, MSD, Pfizer, Jannsen, Novartis, Basilea, GSK, Shionogi, AbbVie, and Advanz Pharma and a grant support from Gilead Science, Pfizer, GSK, MSD, and Pharmamar.. AS has received honoraria for talks on behalf of Merck Sharp and Dohme, Pfizer, Novartis, and Angelini, as well as grant support from Pfizer. JM has received honoraria for talks on behalf of Merck Sharp and Dohme, Pfizer, Novartis, and Angelini. OP has received honoraria for talks on behalf of MSD and Qiagen and expertise for Sanofi.

### Peer Review

The peer review history for this article is available at https://www.webofscience.com/api/gateway/wos/peer‐review/10.1111/irv.13264.

## Supporting information


**Figure S1a.** Flow chart of treatment algorithm.

## Data Availability

Database used for this study will be shared by the corresponding authors under reasonable request.
