# Supplementary figures and images for: Real‐Life Comparison of Antivirals for SARS‐CoV‐2 Omicron Infection in Patients With Hematologic Malignancies
Source: Influenza Other Respir Viruses. 2024 Mar 11;18(3):e13264. doi: 10.1111/irv.13264 (PMC10928260; doi:10.1111/irv.13264)

Figure 1a. Flow chart of the treatment algorithm.


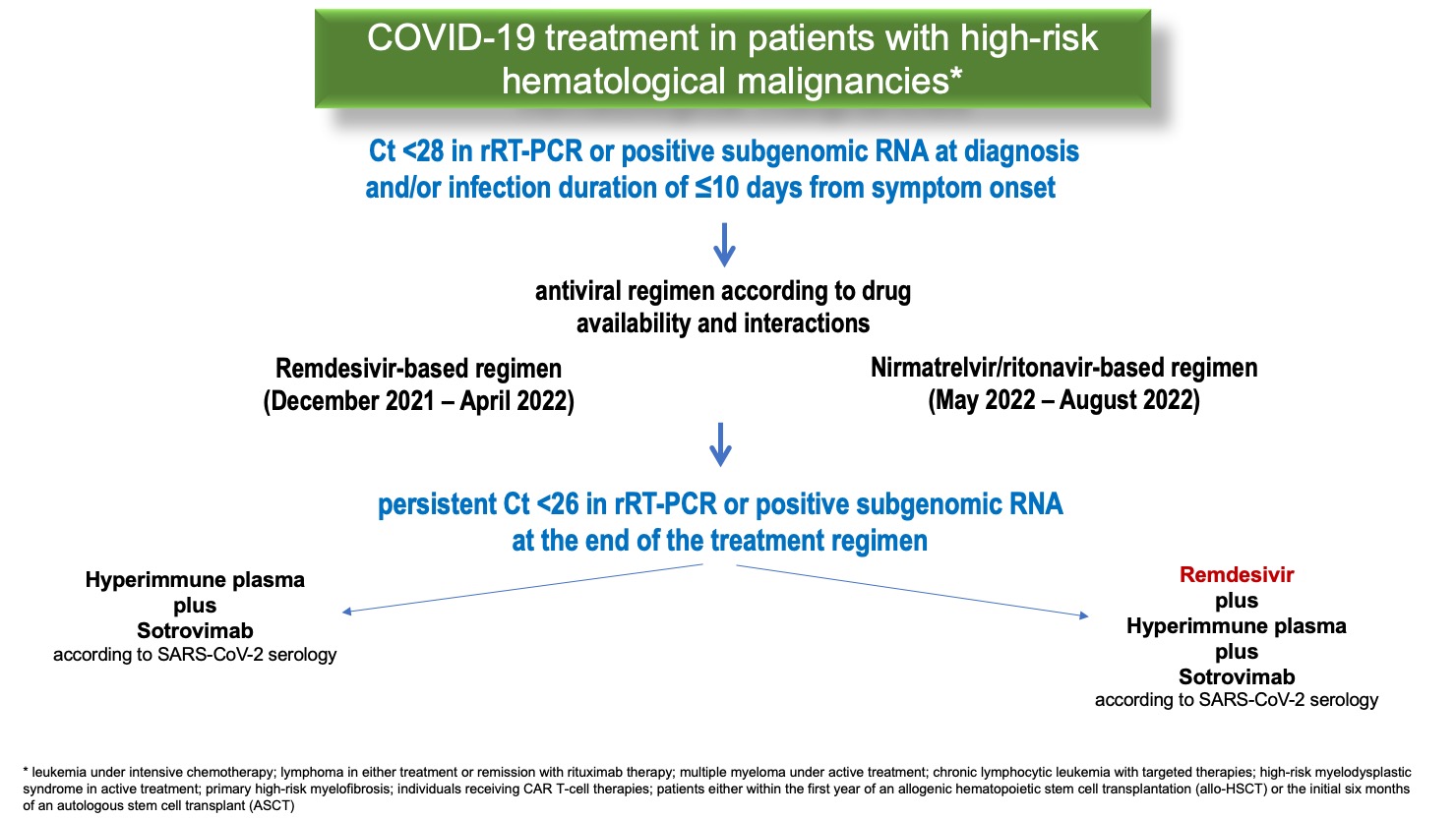

Supplement: Supplementary file 1 — Figure S1a. Flow chart of treatment algorithm. [file IRV-18-e13264-s001.docx]
